# Paclitaxel-incorporated nanoparticles using block copolymers composed of poly(ethylene glycol)/poly(3-hydroxyoctanoate)

**DOI:** 10.1186/1556-276X-9-525

**Published:** 2014-09-24

**Authors:** Hyun Yul Kim, Je Ho Ryu, Chong Woo Chu, Gyung Mo Son, Young-IL Jeong, Tae-Won Kwak, Do Hyung Kim, Chung-Wook Chung, Young Ha Rhee, Dae Hwan Kang, Hyung Wook Kim

**Affiliations:** 1 Department of Surgery, National Research & Development Center for Hepatobiliary Cancer, Pusan National University Yangsan Hospital, Gyeongnam 626-770, Republic of Korea; 2 Biomedical Research Institute, Pusan National University Hospital, Pusan 602-739, Republic of Korea; 3 Department of Microbiology, Chungnam National University, Daejeon 305-764, Korea; 4 National Research & Development Center for Hepatobiliary Cancer, Pusan National University Yangsan Hospital, Gyeongnam 626-770, Republic of Korea; 5 Department of Internal Medicine, Pusan National University Yangsan Hospital, Gyeongnam 626-770, Republic of Korea

**Keywords:** Nanoparticles, Block copolymer, Poly(3-hydroxyoctanoate), Drug delivery

## Abstract

Block copolymers composed of poly(3-hydroxyoctanoate) (PHO) and methoxy poly(ethylene glycol) (PEG) were synthesized to prepare paclitaxel-incorporated nanoparticle for antitumor drug delivery. In a ^1^H-NMR study, chemical structures of PHO/PEG block copolymers were confirmed and their molecular weight (M.W.) was analyzed with gel permeation chromatography (GPC). Paclitaxel as a model anticancer drug was incorporated into the nanoparticles of PHO/PEG block copolymer. They have spherical shapes and their particle sizes were less than 100 nm. In a ^1^H-NMR study in D_2_O, specific peaks of PEG solely appeared while peaks of PHO disappeared, indicating that nanoparticles have core-shell structures. The higher M.W. of PEG decreased loading efficiency and particle size. The higher drug feeding increased drug contents and average size of nanoparticles. In the drug release study, the higher M.W. of PEG block induced the acceleration of drug release rate. The increase in drug contents induced the slow release rate of drug. In an antitumor activity study *in vitro*, paclitaxel nanoparticles have practically similar anti-proliferation activity against HCT116 human colon carcinoma cells. In an *in vivo* animal study using HCT116 colon carcinoma cell-bearing mice, paclitaxel nanoparticles have enhanced antitumor activity compared to paclitaxel itself. Therefore, paclitaxel-incorporated nanoparticles of PHO/PEG block copolymer are a promising vehicle for antitumor drug delivery.

## Background

Nanoparticles have been extensively investigated in the biomedical field as a drug delivery vehicle due to their properties such as small particle size less than 1,000 nm, favorable biodistribution, and drug targeting possibility [1,2]. Among them, core-shell type nanoparticles have great potentials in the antitumor drug delivery system since they have intrinsic properties, i.e., hydrophobic core as a drug reservoir and hydrophilic outershell as a protective layer [2,3]. They have superior properties such as extended blood circulation time of drug, passive targeting of solid tumor, dissolution of hydrophobic drugs, and enhanced biodegradation.

Poly(3-hydroxyoctanoate) (PHO) is one of the polyesters derived from bacterial source [4,5]. PHO has attractive properties as a biomedical material such as low melting temperature and crystallinity and excellent elastomeric and mechanical properties compared to other kinds of polyhydroxyalkanoates [4-6]. The drawback of PHO is slower degradation rate and higher hydrophobic properties than other kinds of PHAs [6]. Therefore, various chemical modifications of PHO have been attempted to endow hydrophilic properties and to improve mechanical properties as a biomedical material [5,7].

In this study, we synthesized block copolymers composed of PHO/PEG to make core-shell-typed nanoparticles and to fabricate suitable vehicles for antitumor drug delivery. Since PHO has hydrophobicity, it could be consisted in the core of the nanoparticles while PEG must be hydrated to the direction of aqueous environment. Paclitaxel was selected as an anticancer agent due to its poor aqueous solubility. Characteristics of nanoparticles of PHO/PEG block copolymers were analyzed and antitumor activity of drug-incorporated nanoparticles was studied *in vitro*/*in vivo*.

## Methods

### Materials

PHO was produced by *Pseudomonas oleovorans* ATCC 29347 (American Type Culture Collection (ATCC), Manassas, VA 20110, USA) as reported previously [4] and purified from the lyophilized cells by extraction with hot chloroform [8]. The purified PHO analyzed by gas chromatography was consisted of 7% 3-hydroxyhexanoate and 93% 3-hydroxyoctanoate. Methoxy poly(ethylene glycol)-amine (molecular weight (M.W.) = 2,000, 5,000, 12,000 g/mol) was purchased from Sunbio Co. Ltd., Cheonan, Korea. Fluorescein isothiocyanate (FITC), *N*,*N*′-dicyclohexyl carbodiimide (DCC), and *N*-hydroxysuccinimide (NHS) were purchased from Aldrich Chemical Company (St Louis, MO, USA). The dialysis membranes with molecular weight cut-offs (MWCOs) of 12,000 and 8000 g/mol were purchased from Spectra/PorTM dialysis membrane (Spectrum Laboratories Inc, Rancho Dominguez, CA, USA). Dichloromethane (DCM), dimethylformamide (DMF), tetrahydrofuran (THF), methanol, and acetone were of extra pure grade.

### Synthesis of PHO/PEG block copolymers

**Figure 1:**
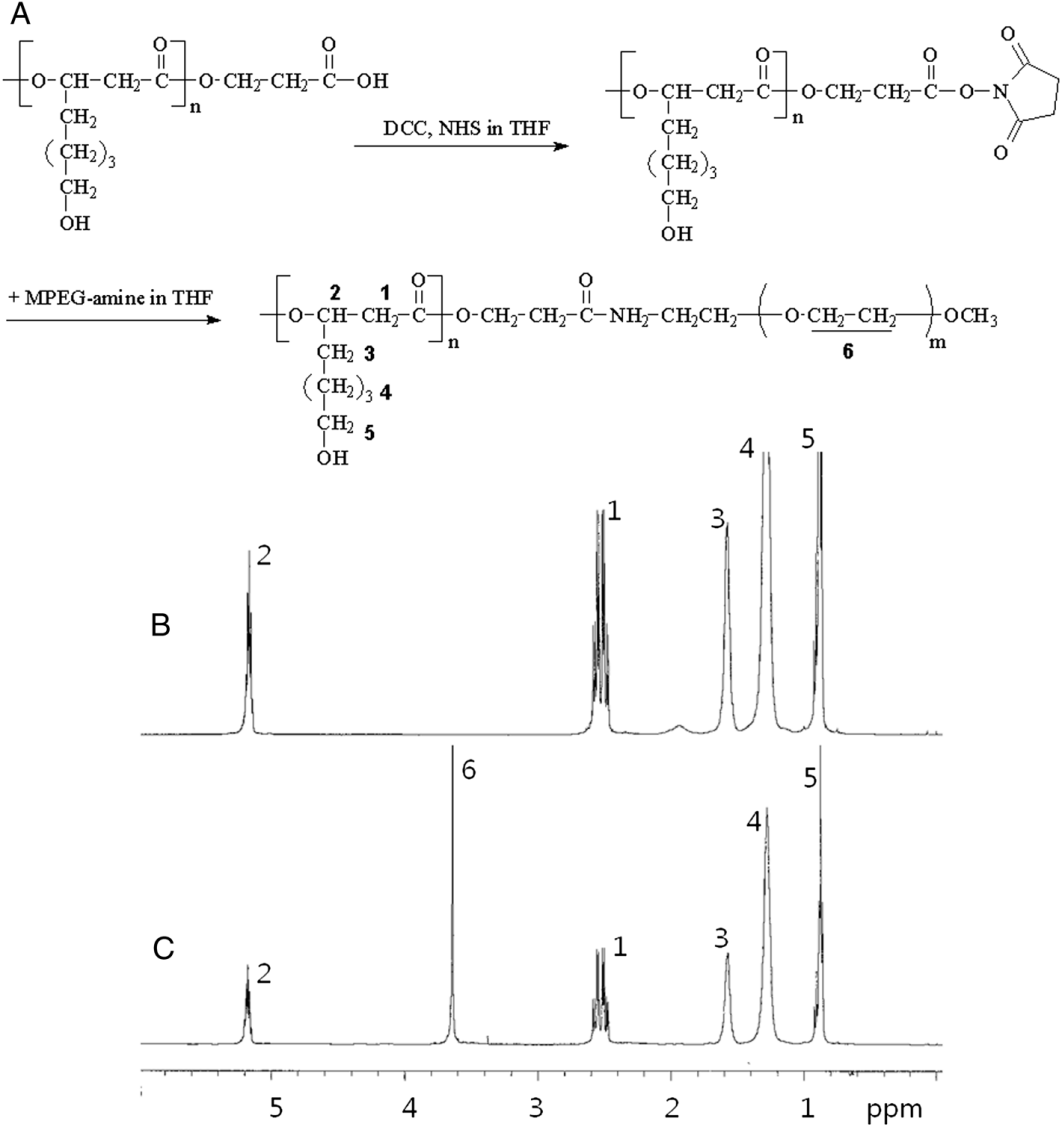
**Synthesis scheme (A) and **^**1**^**H-NMR spectra of copolymers (B, C).** Synthesis scheme of PHO/PEG block copolmyers **(A)**. ^1^H-NMR spectra of PHO **(B)** and PHO/PEG 12 K **(C)** in CDCl_3_.

Five hundred grams of PHO were dissolved in 20 ml of THF. Ten milligrams of DCC and 5.5 mg of NHS was added to this solution and magnetically stirred for 6 h. After that, the solution was filtered using filter paper to remove by-products and the solvent was evaporated using rotary evaporator following precipitation into methanol. Precipitates were harvested and dried under reduced pressure for 2 days to obtain NHS-activated PHO as shown in Figure 1. Five hundred milligrams of NHS-activated PHO was dissolved in 20 ml of THF and 2 equivalents of MPEG amine were added. This solution was stirred for 24 h and then precipitated into excess amount of methanol three times. Precipitates were harvested and dried under vacuum for 3 days.

### Characterization of PHO/PEG block copolymer

Characterization of the synthesized polymers was performed in CDCl_3_ by 500 mHz ^1^H-NMR spectroscopy (500 mHz superconducting FT-NMR spectrometer, Varian Unity Inova 500 MHz NB High Resolution FT NMR; Varian Inc, Santa Clara, CA, USA). Molecular weight of polymers was measured with the gel permeation chromatography (GPC) system (Waters 2690D-2410, Waters, MA, USA) as described previously [5]. GPC was equipped with a Waters 6000 solvent delivery system, RI detector, and a Rheodyne injector. Phenogel columns of 500, 10^3^, 10^4^, and 10^5^ Å were used. A standard curve was established with standard polystyrene samples.

### Preparation of paclitaxel-incorporated nanoparticles using PHO/PEG block copoylmer

PHO/PEG block copolymer was dissolved in 5 ml of THF and then paclitaxel dissolved in 2 ml of DMF was added. This solution was slowly dropped into 10 ml of cold deionized water for 15 min and then stirred magnetically for 60 min at 4°C. After that, this solution was dialyzed using a dialysis tube (MWCO = 12,000 g/mol) against deionized water for 1 day. To remove solvent, deionized water was exchanged at every 2-h intervals. After that, dialyzed solution was used for analysis or lyophilization. Empty nanoparticles were prepared in the absence of paclitaxel.

Drug content and loading efficiency were measured using a UV-1201 spectrophotometer (Shimadzu Co. Ltd., Kyoto, Japan) at 273 nm. Empty nanoparticles were used as a blank.

Morphology of the polymeric micelles was observed using a transmission electron microscope (TEM, JEOL JEM-2000 FX II, JEOL, Akishima-shi, Japan). One drop of nanoparticle solution containing phosphotungstic acid 0.05% (*w*/*w*) was placed onto a carbon film coated on a copper grid for TEM. Observation was done at 80 kV. The particle size of the nanoparticles was measured with dynamic laser scattering (DLS-7000, Otsuka Electronics Company, Osaka, Japan).

### Drug release study

The release experiment was carried out *in vitro* as follows: Paclitaxel-incorporated nanoparticle solution was prepared as described above. After the dialysis procedure, the volume of nanoparticle solution was adjusted to 50 ml and then 5 ml of this solution was introduced into a dialysis tube (MWCO = 12,000 g/mol) and then dialysis tube was introduced into a 200-ml bottle with 95 ml of phosphate-buffered saline (PBS, 0.1 M, pH 7.4, 0.1% (*w*/*v*) Tween 80). This bottle was placed in a shaking incubator with stirring speed of 100 rpm at 37°C. At specific time intervals, the media were taken for analysis of drug concentration. After that, the whole media was replaced with fresh PBS to prevent drug saturation. The concentration of the paclitaxel released into PBS was evaluated using a high-performance liquid chromatography (HPLC, PerkinElmer, Waltham, MA, USA) using Phenomenex Sphereclone 5 micro ODS(2) 250 mm × 4.6 mm column and 75% methanol as a moving phase. The Flexar HPLC system (PerkinElmer, Waltham, MA, USA) was equipped with a Solvent Manager 5-CH degasser, an autosampler, a quaternary LC pump, a column oven, and a UV-visible detector. The flow rate was set to 1.5 ml/min and the temperature at 50°C. The UV detector (226 nm) was used for the measurement. To prepare the sample for the measurement of HPLC, paclitaxel was dissolved in ethanol, making 20 μl for injection.

### Antitumor activity of paclitaxel-incorporated nanoparticles in vitro

Antitumor activity of paclitaxel-incorporated nanoparticles was evaluated using HuCC-T1 human cholangiocarcinoma cells. HCT116 cells were seeded at a density of 2 × 10^3^ cells per well in 96-well plates with 100 μl of RPMI1640 supplemented with 10% FBS and incubated overnight at 37°C and 5% CO_2_ atmosphere. After that, free paclitaxel, paclitaxel-incorporated nanoparticles and empty nanoparticles were added to 96-well plates at 100 μl. For paclitaxel treatment, paclitaxel dissolved in DMSO was diluted 1,000 times with RPMI1640 supplemented with 10% FBS and added to cells. For nanoparticle treatment, paclitaxel-incorporated nanoparticles were diluted with RPMI1640 supplemented with 10% FBS and treated to cells. Controls were treated with 0.1% *v*/*v* of dimethyl sulfoxide. For the testing of cellular cytotoxicity of empty nanoparticles, they were diluted with serum-free RPMI1640 media and sterilized with a 0.8-μm syringe filter and added to 2 × 10^4^ HCT116 cells. Viable cells were evaluated with MTT cell proliferation assay as described previously [9]. After 3 days of incubation, 30 μl of MTT (5 mg/ml) was added to the 96-well plates and incubated for 4 h. The formazan crystals formed were solubilized with SDS solution (100 μl of SDS-HCl solution (SDS 10% *w*/*v*, 0.01 M HCl)) and the absorbance (560-nm test/630-nm reference) was determined using an automated computer-linked microplate reader (Infinite M200 Pro microplate reader, Molecular Device Company, Sunnyvale, CA, USA). Each measurement of drug concentration was obtained as the mean value of eight wells. The amount of formazan present is proportional to the number of viable cells, because only living cells will reduce MTT to blue formazan. The results were expressed as a percentage of the absorbance present in the drug-treated cells compared with that in the control cells.

### Apoptosis/necrosis analysis

Apoptosis and necrosis of HCT116 cells were analyzed by flow cytometry [9]. FITC-annexin V and PI were employed to analyze apoptosis and necrosis of HCT116 cells, respectively. Cells (1 × 10^6^ cells) were exposed to paclitaxel or paclitaxel-incorporated nanoparticles in serum-free RPMI1640 media for 48 h, and cells were washed with phosphate-buffered saline (PBS). The cells were harvested by trypsinization, and cell pellets were resuspended in a binding buffer (pH 7.4, 10 mM HEPES, 150 mM NaCl, 5 mM KCl, 1 mM MgCl_2_, 1.8 mM CaCl_2_) containing FITC-annexin V (1 μg/ml) and then further incubated for 30 min. Ten minutes prior to termination of incubation, PI (10 μg/ml) were added to stain necrotic cells under dark conditions. Following that, the cells were immediately analyzed using a FACScan flow cytometer (Becton Dicknson Biosciences, San Jose, CA, USA).

### Antitumor activity of paclitaxel-incorporated nanoparticles against animal tumor xenograft

The tumor xenograft model was employed to assess antitumor activity of paclitaxel or paclitaxel-incorporated PA nanoparticles. HCT116 cells (5 × 10^6^) were implanted subcutaneously in the backs of mice (Athymic nude BALB/c male mice, 20 g). When the diameter of tumor mass was approximately 2 ~ 3 mm (10 days after), paclitaxel or paclitaxel-incorporated nanoparticles were injected intravenously via tail vein of the mouse (paclitaxel dose = 10 mg/kg). The treatment group was divided as follows; control - 200 μl of PBS (0.01 M, pH 7.4); paclitaxel treatment - paclitaxel dissolved in Cremophor® EL/ethyl alcohol (5:5, (*v*/*v*)) solution were mixed with aqueous solution of empty nanoparticles; paclitaxel-nanoparticles prepared from PHO/PEG 5 K - lyophilized nanoparticles were reconstituted in deionized water and sterilized with a 0.8-μm syringe filter. Five mice were used for each treatment group. At intervals of 5 days, length and width of solid tumor was measured and tumor volume was calculated with the following equation: Tumo volume (mm^3^) = Length × Width^2^. Tumor mass was taken from the back of mice and the weight of tumor was compared between each of the treatment groups 2.5 weeks after drug injection. All animal experiments followed the guidelines of the Committee for the Care and Use of Laboratory Animals of Pusan National University.

### Statistical analysis

Results of animal experiments were expressed as average ± standard deviation, and statistical significance was evaluated using the Student's *t* test (SigmaPlot program version 11.2, Systat software, Inc., CA 95110, USA). A *p* value <0.05 was considered significant.

## Results

### Characterization of PHO/PEG block copolymer

As reported previously, PHO was produced from *P. oleovorans* ATCC 29347 using octanoate-supplemented mineral medium [8]. PHO was purified from lyophilized cells by extraction with hot chloroform because cell lysates were not soluble in chloroform and PHO can be solved in it. The purified PHO was composed of 3-hydroxyhexanoate (7%) and 3-hydroxyoctanoate (93%) [8]. To synthesize block copolymers composed of PHO and PEG, carboxylic end group of PHO was activated using DCC and NHS, and then PHO-NHS was reacted with various M.W. of MPEG-amine (Figure 1A). Since MPEG-amine is soluble in methanol, reactants were precipitated into methanol. Final product was analyzed using ^1^H-NMR spectroscopy in CDCl_3_. The chemical structures of PHO/PEG block copolymer were shown in Figure 1B,C. As shown in Figure 1B, typical ^1^H-NMR spectra of the PHO homopolymer showed specific peaks around 0.9, 1.3, 1.6, 2.56, and 5.2 ppm, respectively. In the PHO/PEG block copolymer as shown in Figure 1C, the ethylene peak of PEG appeared at 3.65 ppm. M.W. of PHO homopolymer and PHO/PEG block copolymer were analyzed using GPC as shown in Table 1. As shown in Table 1, the M.W. of PHO/PEG block copolymer was increased as much as PEG M.W. at each sample even though the gap of M.W. was not exactly the same. These results indicated that MPEG was successfully attached to the carboxylic end of the PHO block.

**Table 1:** Characterization of PHO/PEG block copolymers

	**M.W. of PEG (g/mol)**^**a**^	**M.W. by GPC (g/mol)**			**Particle size (weight average, nm)**
		**M**_**w**_	**M**_**n**_	**PDI**^**b**^	
PHO	-	57,800	51,900	1.11	97.2
PHO/PEG 2 K	2,000	60,200	53,200	1.13	78.7
PHO/PEG 5 K	5,000	63,100	60,900	1.04	72.6
PHO/PEG 12 K	12,000	70,500	65,300	1.08	62.8

^a^M.W. of PEG was from the manufacturer's data; ^b^PDI (polydispersity index) was M_w_/M_n_.

### Characterization of paclitaxel-incorporated nanoparticles

**Figure 2:**
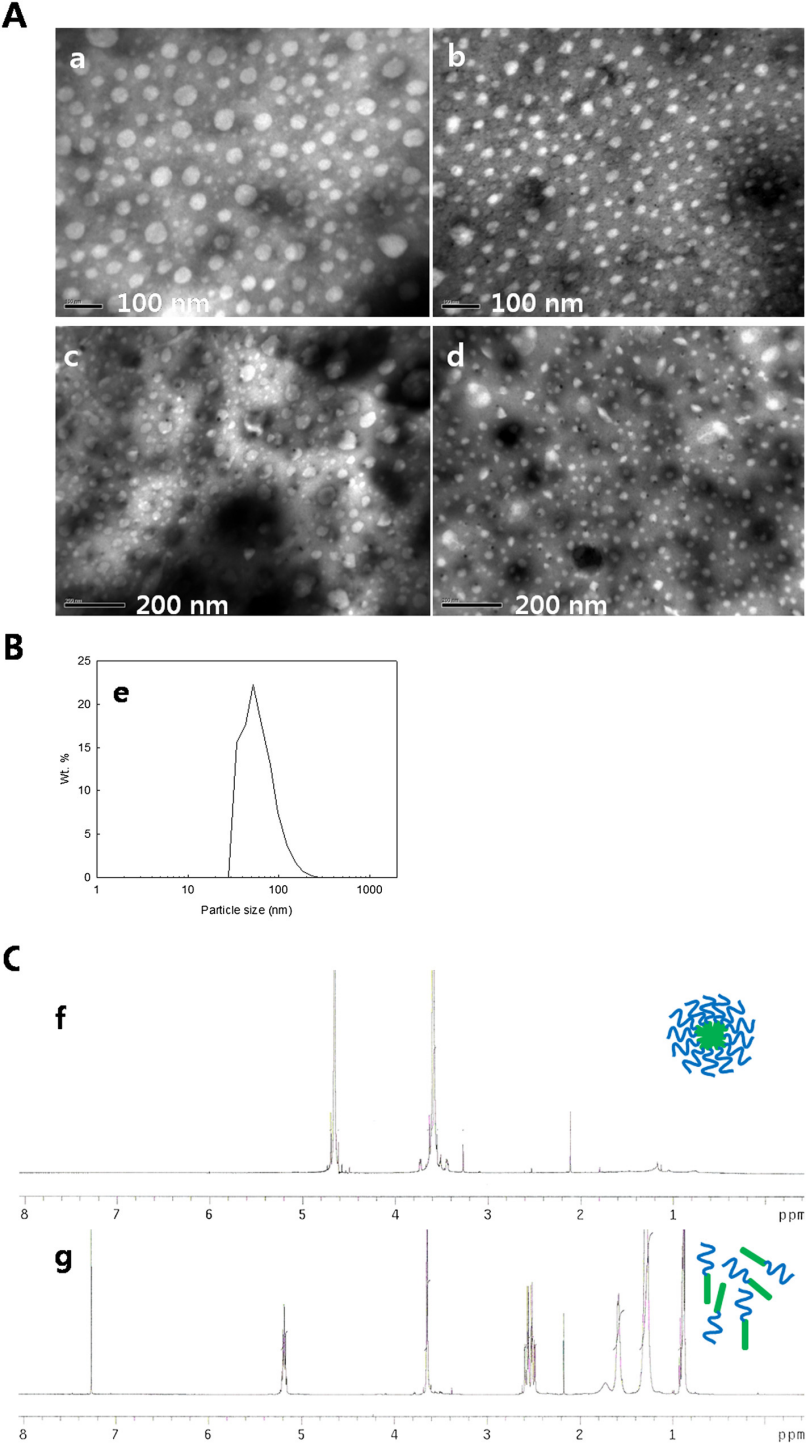
**TEM photos (A), typical size distribution (B), and **^**1**^**H-NMR spectra of nanoparticles (C). (A)** TEM photos of nanoparticles of PHO/PEG block copolymer. PHO (a), PHO/PEG 2 K (b), PHO/PEG 5 K (c), and PHO/PEG 12 K (d). **(B)** Typical particle size distribution (e) of PHO/PEG 12 K nanoparticles. **(C) **^1^H-NMR spectra of nanoparticles of PHO/PEG block copolymer (PHO/PEG 12 K) in D_2_O (f) and CDCl_3_ (g).

Nanoparticles of PHO/PEG block copolymers were fabricated by nanoprecipitation-diafiltration method, i.e. block copolymer dissolved in solvent was precipitated into deionized water to make nanoparticles and solvent was removed by dialysis membrane. To confirm whether or not nanoparticles were formed, nanoparticle solution was analyzed using dynamic light scattering and TEM. As shown in Figure 2A, nanoparticles were successfully fabricated at all of the polymers and their average particle sizes were less than 100 nm (Figure 2B). Furthermore, particle sizes were decreased according to the increase of M.W. PEG block (Table 1), indicating that hydrophilic nature of PEG block might have increased hydrophilicity of block copolymer by higher M.W. of PEG since PEG is a water-soluble polymer. The higher M.W. of PEG in the block copolymer is known to cause decreased particle size, increased colloidal stability, and increased biological properties [10]. Since block copolymers composed of hydrophilic and hydrophobic blocks are normally formed nanoparticles with core-shell structure, nanoparticles of PHO/PEG block copolymer were examined using ^1^H-NMR spectroscopy with D_2_O. As shown in Figure 2C, specific peaks of PEG were only observed when nanoparticles were distributed in D_2_O (Figure 2C(f)). When nanoparticles were added to CDCl_3_, however, specific peaks both PHO and PEG appeared as shown in Figure 2C(g). These results indicated that nanoparticles of PHO/PEG block copolymers are formed core-shell structures, i.e., PEG block consisted an outer shell of nanoparticles while PHO block consisted a solid inner core of nanoparticles.

Paclitaxel was loaded into nanoparticles by nanoprecipitation and dialysis procedure. Even though direct dialysis was also possible to form nanoparticles, it also induced the increase in particle size higher than 300 nm (data not shown) while nanoprecipitation resulted in reduced particle size. Because DMF is a non-volatile solvent and hard to remove by evaporation, the remaining solvent was removed by dialysis. The results of paclitaxel-incorporated nanoparticles of PHO/PEG block copolymers are summarized in Table 2. As shown in Table 2, the higher M.W. of PEG induced the decrease in the amount of loaded drug and loading efficiency as well. Furthermore, drug entrapment into nanoparticles induced the increase in particle size compared that into empty nanoparticles. The higher the drug feeding ratio, the higher the drug contents and average size of nanoparticles.

**Table 2:** Characterization of paclitaxel-incorporated nanoparticles of PHO/PEG block copolymer

	**Polymer/drug (mg/mg)**	**Drug contents (%, *w*/ *w *)**		**Particle size (weight average, nm)**
		**Theoretical**	**Experimental**	
PHO	45/5	10	7.1	115.1
PHO/PEG 2 K	45/5	10	6.8	103.1
PHO/PEG 5 K-1	45/5	10	6.5	97.6
PHO/PEG 5 K-2	40/10	20	11.9	110.8
PHO/PEG 12 K	45/5	10	6.6	82.6

**Figure 3:**
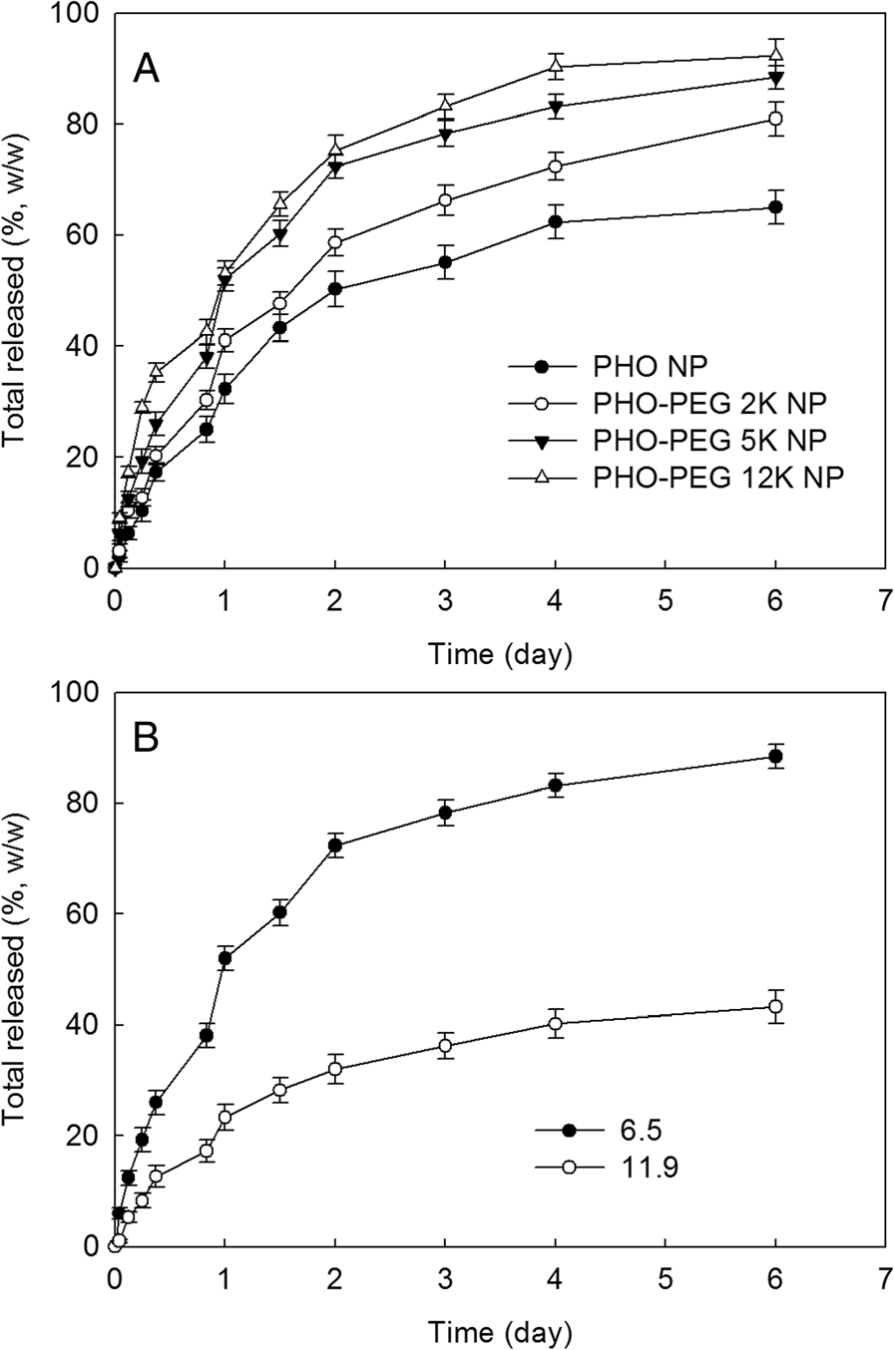
**Drug release from paclitaxel-incorporated nanoparticles of PHO/PEG block copolymers.** The effect of PEG length **(A)** and drug contents **(B)**. NP, nanoparticles.

Figure 3 shows paclitaxel release from the nanoparticles of PHO/PEG block copolymer. As shown in Figure 3A, the higher M.W. of PEG block induced increase of faster drug release rate than the lower M.W. of PEG block. The higher PEG M.W. is known to accelerate drug release rate [10]. Initial burst release of drug was observed until 12 h and then drug release profile was relatively continuous manner. Furthermore, increased drug contents induced slower release rate of drug as shown in Figure 3B. Since paclitaxel is hydrophobic drug, hydrophobic interactions might be increased at higher drug content and this phenomenon caused decreased drug release rate. At higher drug contents, hydrophobic drug can be aggregated in the solid core of the nanoparticles and aggregated drug release slowly from the nanoparticles [2,3]. Furthermore, larger particle size at higher drug loading might be the one of reason for slower release [11].

### Antitumor activity of paclitaxel-incorporated nanoparticles

**Figure 4:**
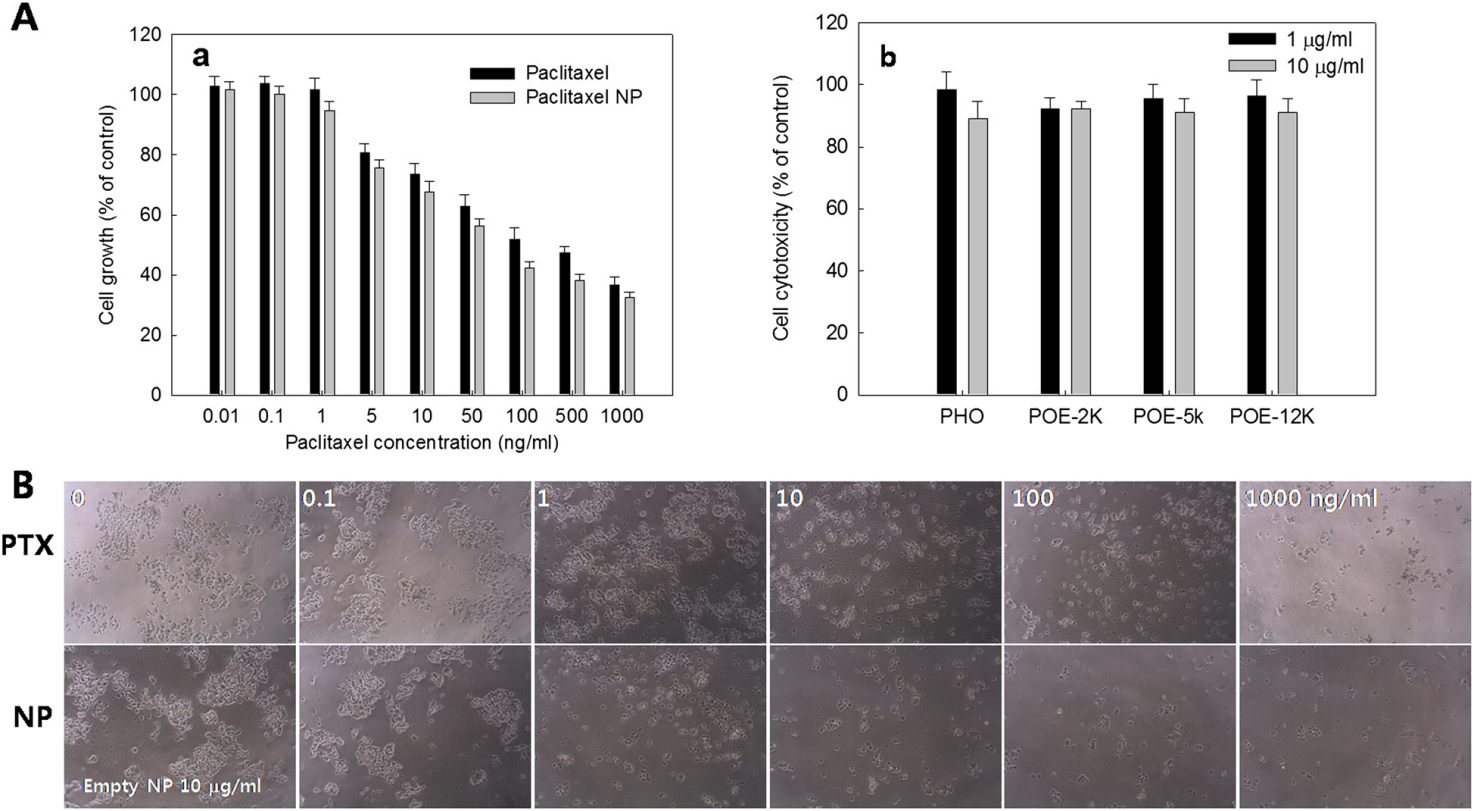
**Growth inhibition and cell cytotoxicity (A) and cell morphology (B) of nanoparticles. (A)** Growth inhibition of paclitaxel or paclitaxel-incorporated PHO/PEG 5 K nanoparticles (NP) against HCT116 cells (a). Survivability of HCT116 was measured by MTT assay. Empty nanoparticles were treated to tumor cells for 2 days (b). **(B)** Cell morphology with treatment of paclitaxel, empty nanoparticles, and paclitaxel-incorporated nanoparticles. For 0 μg/ml of nanoparticle treatment, 10 μg/ml of empty nanoparticles were treated to cells. Paclitaxel NP, paclitaxel-incorporated nanoparticles. Empty NP, empty nanoparticles.

**Figure 5:**
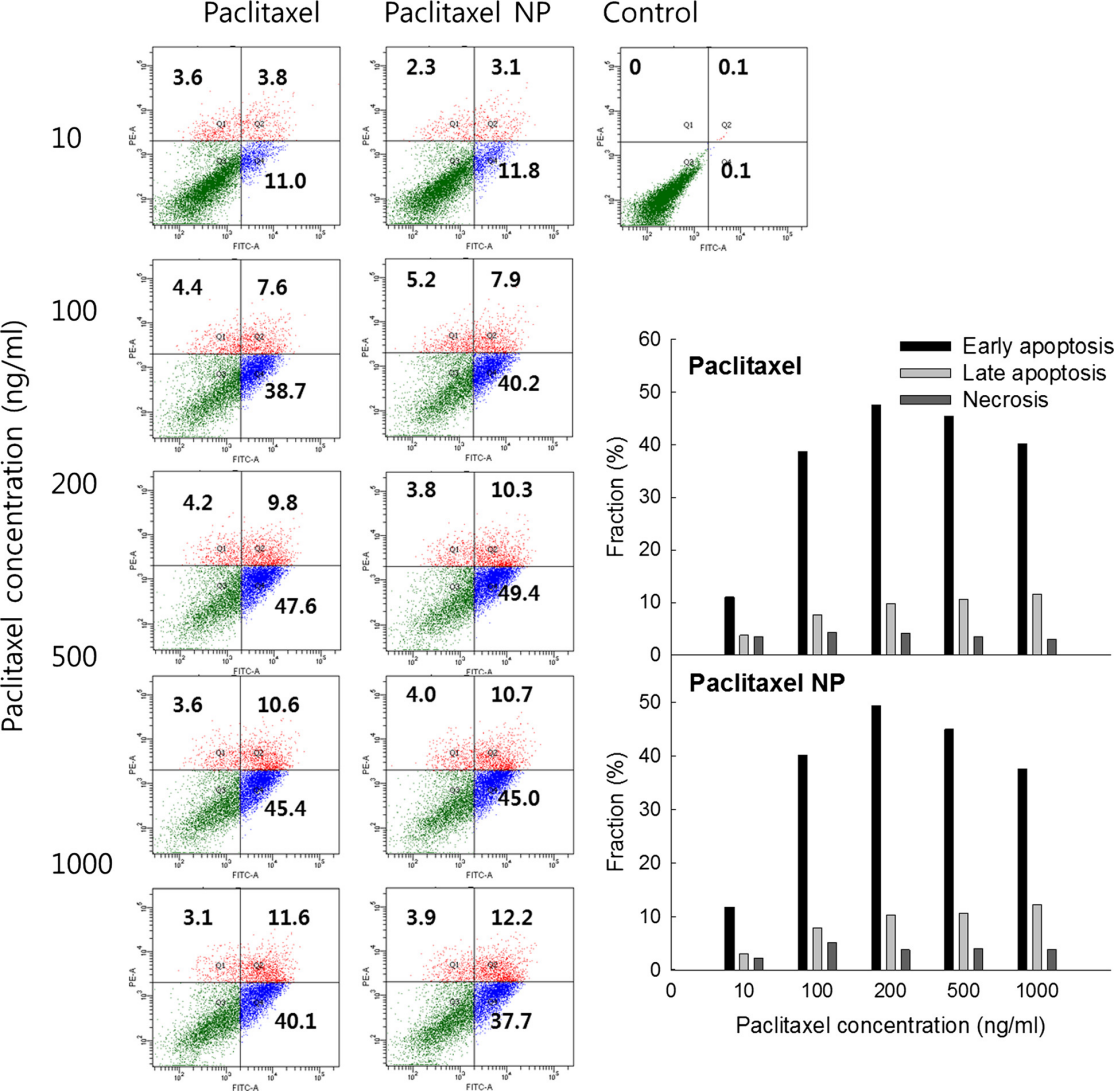
**Flow cytometric analysis of HCT116 cells.** Annexin V was used for apoptosis analysis and PI was used for necrosis analysis. HCT116 cells with treatment of paclitaxel, empty nanoparticles, and paclitaxel-incorporated nanoparticles (PHO/PEG 5 K).

The antitumor activity of paclitaxel-incorporated nanoparticles of PHO-PEG block copolymer was tested using HCT116 human colon carcinoma cells *in vitro*. As shown in Figure 4A(a), viable cells were decreased according to the exposure time of paclitaxel. Especially, paclitaxel-incorporated nanoparticles showed enhanced antiproliferation activity at the same paclitaxel dose while empty nanoparticles did not show anti-proliferation effect against tumor cells as shown in Figure 4A(b). Figure 4B showed cell morphology according to the treatment of paclitaxel or paclitaxel nanoparticles. As shown in Figure 4B, cell density was decreased according to the increase of paclitaxel dose. Cells were crowded at a lower dose of paclitaxel while they were scattered at a higher concentration of paclitaxel.Figure 5 shows the apoptosis/necrosis analysis of HCT116 cells with treatment of paclitaxel and paclitaxel nanoparticles. As shown in Figure 5, apoptotic cells were dose-dependently increased and, especially, apoptotic cells with treatment of paclitaxel nanoparticles were practically similar to those treated with paclitaxel even though they were slightly higher. These results also supported those of Figure 4.

**Figure 6:**
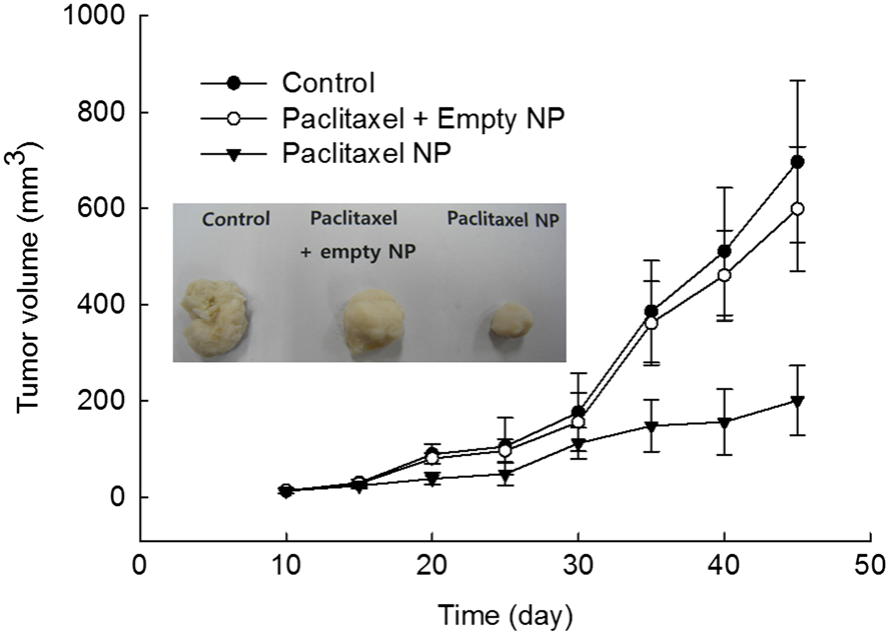
**Tumor mass of HCT116 tumor xenograft-induced nude mice.** Ten days after tumor implantation, drug solution was injected intravenously (Paclitaxel dose 10 mg/kg). Control - PBS (0.1 M, pH 7.4); Paclitaxel + Empty nanoparticle treatment - paclitaxel dissolved in Cremophor EL/ethanol mixture (5/5, *v*/*v*) was diluted ten times with PBS and mixed with aqueous solution of empty nanoparticles (the content of empty nanoparticles in this treatment were adjusted to similar concentration with paclitaxel NP treatment; paclitaxel-incorporated nanoparticles (Paclitaxel NP) - aqueous solution of paclitaxel-incorporated nanoparticles (PHO/PEG 5 K) was sterilized with a 0.8-μm syringe filter.

*In vivo* antitumor activity of paclitaxel-incorporated nanoparticles against HCT116 cells were evaluated with tumor xenograft model using nude mice. As shown in Figure 6, tumor weight with treatment of paclitaxel nanoparticles were significantly lower than that of control or paclitaxel + empty nanoparticles while paclitaxel + empty nanoparticles were not significantly changed tumor mass compared to control. At least, tumor mass of mice treated with paclitaxel nanoparticles were three times smaller than that of paclitaxel + empty nanoparticles. The antitumor activity of paclitaxel at the animal tumor xenograft model might be due to the sustained release function of nanoparticles. These results indicate that paclitaxel-incorporated nanoparticles are a potent anticancer agent compared to conventional carriers.

## Discussion

Nanoparticles are regarded as ideal vehicles for anticancer drug targeting due to their intrinsic physicochemical properties [12]. Even though various active targeting using receptor-ligand interaction strategy have been investigated successively [12-15], passive targeting using plain nanoparticles or PEGylated nanoparticles are still preferentially considered for tumor targeting due to the vasculature permeability of tumor tissue [16,17]. Bae insisted that numerous biological and physiological elimination mechanisms of tumor preferentially decide the fate of nanovehicles prior to their access in the tumor cells [16]. Until the nanocarriers are close to the cell surfaces, no specific interaction between receptor on the tumor cell surface and ligand on the nanoparticle surface happens. At this moment, the preferential translocation of nanoparticles from blood circulation to tumor vasculature (so-called enhanced permeation and retention (EPR) effect) is still considered for a solid tumor targeting [16,17]. Diameter of nanoparticles less than 200 nm are known to have advantages for drug targeting because this size could increase blood circulation by avoiding reticuloendothelial system (RES) and then increase chance to access the microvessel of the tumor tissue [17,18].

In this study, we synthesized PHO/PEG block copolymers and prepared paclitaxel-incorporated core-shell-typed nanoparticles for anticancer drug delivery. There are very few reports on the drug delivery application with PHO in spite of their defined structure and good biocompatibility [6,19-21]. The main drawback of PHO is slow degradation *in vitro* and *in vivo*[6,19]. In a previous research, graft-copolymerization with PEG accelerated hydrolytic degradation of polymers [6]. PHO/PEG block copolymer was synthesized by the attachment of PEG to the carboxylic end of PHO. We prepared PHO/PEG block copolymers by varying M.W. of PEGs and its effect on the physicochemical properties of nanoparticle formation. We found that higher M.W. of PEG decreased particle size of nanoparticles even though it slightly decreased drug loading contents (in Tables 1 and 2).

For antitumor drug delivery, paclitaxel was selected as an anticancer drug because it is commonly used in the clinics and has strong hydrophobic property (aqueous solubility is less than 0.3 μg/ml) and it has a specific interaction with serum proteins [22]. Due to its poor aqueous solubility, specific surfactant/solvent mixtures (Cremophor EL/ethanol mixture) are commercially used. However, due to the intrinsic toxicity of specific surfactant, various formulations for enhancing aqueous solubility of paclitaxel have been investigated such as nanoparticles, liposomes, and polymeric micelles [23]. Since PEG has a protective effect for the adsorption of protein and macrophage cells, PEGylation of nanocarriers may enhance blood circulation time of nanocarriers, unwanted content with serum protein/macrophage cells, and then chance to contact tumor tissues *in vivo*.

Paclitaxel was incorporated into the nanoparticles by nanoprecipitation and dialysis procedure. The size of paclitaxel nanoparticles was slightly increased compared to that of empty nanoparticles. Even though the diameter of nanoparticles was increased with paclitaxel loading, the average size of nanoparticles did not exceed 200 nm. Furthermore, paclitaxel was slowly released from the nanoparticles for over 6 days. In a drug release study, diffusion from nanoparticles must be the preferential mechanism rather than the degradation of polymer because PHO itself has very slow degradation kinetics [6,19]. Nanoparticles showed practically similar anticancer activity against tumor cells as shown in Figure 4. Furthermore, nanoparticles showed practically similar effect on the apoptosis and necrosis of tumor cells as shown in Figure 5. Antitumor activity of paclitaxel nanoparticles *in vivo* was evaluated using HCT116 tumor-bearing mice as shown in Figure 6. As shown in Figure 6, paclitaxel nanoparticles significantly inhibited growth of tumor mass compared to the control and the paclitaxel plus empty nanoparticles. Even though nanoparticles have no practical advantages in anticancer activity *in vitro*, they properly suppressed growth of tumor mass *in vivo* the tumor xenograft model as shown in Figure 6 while paclitaxel itself seemed to have minimal anticancer activity. The EPR effect of the nanoparticles can be considered to explain these results, i.e., nanoparticles properly entered into the cells and killed the tumor cells [16,17]. Otherwise, paclitaxel itself could be absorbed to the serum protein in the blood and rapidly cleared from blood circulation. Then, these factors can induce decrease in opportunities for tumor targeting [22]. Therefore, paclitaxel nanoparticles formed by PHO/PEG block copolymer are a promising vehicle for antitumor drug delivery.

## Conclusions

Paclitaxel-incorporated nanoparticles were prepared using PHO/PEG block copolymers for antitumor drug delivery. Paclitaxel-incorporated nanoparticles of PHO/PEG block copolymer have spherical morphology with small particle size less than 100 nm. In a ^1^H-NMR study in D_2_O, the core-shell structure of PHO/PEG nanoparticles was confirmed. The higher M.W. of PEG decreased loading efficiency of drug and particle size. The higher drug feeding resulted in increased drug contents and average size of nanoparticles. The higher M.W. of PEG block accelerated drug release rate while higher drug contents delayed the release rate of drug. In an antitumor activity study using HuCCT1 cells *in vitro*, paclitaxel-incorporated nanoparticles have similar antiproliferation activity against HCT116 tumor cells. However, paclitaxel nanoparticles showed enhanced antitumor activity against HCT116 tumor cells of mice *in vivo*.

## Competing interests

The authors declare that they have no competing interests.

## Authors’ contributions

HYK carried out the fabrication and characterization of nanoparticles. JHR, CWC, and GMS participated in the morphological observation, particle size analysis, and spectroscopical analysis of polymers and nanoparticles. TWK, DHK, CWC, and YHR contributed to the synthesis of microbial polymers. YIJ designed and synthesized block copolymers. DHK and HWK conceived the study and coordination. All authors read and approved the final manuscript.

## References

[B1] Kreuter J Drug targeting with nanoparticles Eur J Drug Metab Pharmacokinet 1994 19 253 256 10.1007/BF03188928 7867668

[B2] Gref R Minamitake Y Peracchia MT Trubetskoy V Torchilin V Langer R Biodegradable long-circulating polymeric nanospheres Science 1994 263 1600 1603 10.1126/science.8128245 8128245

[B3] Jeong YI Cheon JB Kim SH Nah JW Lee YM Sung YK Akaike T Cho CS Clonazepam release from core-shell type nanoparticles in vitro J Control Release 1998 51 169 178 10.1016/S0168-3659(97)00163-6 9685914

[B4] Kim DY Kim YB Rhee YH Evaluation of various carbon substrates for the biosynthesis of polyhydroxyalkanoates bearing functional groups by Pseudomonas putida Int J Biol Macromol 2000 28 23 29 10.1016/S0141-8130(00)00150-1 11033174

[B5] Kim HW Chung CW Rhee YH UV-induced graft copolymerization of monoacrylate-poly(ethylene glycol) onto poly(3-hydroxyoctanoate) to reduce protein adsorption and platelet adhesion Int J Biol Macromol 2005 35 47 53 10.1016/j.ijbiomac.2004.11.007 15769515

[B6] Kim HW Chung CW Hwang SJ Rhee YH Drug release from and hydrolytic degradation of a poly(ethylene glycol) grafted poly(3-hydroxyoctanoate) Int J Biol Macromol 2005 36 84 89 10.1016/j.ijbiomac.2005.03.016 15936069

[B7] Arkin AH Hazer B Borcakli M Chlorination of poly(3-hydroxy alkanoates) containing unsaturated side chains Macromolecules 2000 33 3219 3223 10.1021/ma991535j

[B8] Kim DY Jung SB Choi GG Kim YB Rhee YH Biosynthesis of polyhydroxyalkanoate copolyester containing cyclohexyl groups by Pseudomonas oleovorans Int J Biol Macromol 2001 29 145 150 10.1016/S0141-8130(01)00144-1 11589966

[B9] Jeong YI Kim DH Chung CW Yoo JJ Choi KH Kim CH Ha SH Kang DH Doxorubicin-incorporated polymeric micelles composed of dextran-b-poly(DL-lactide-co-glycolide) copolymer Int J Nanomedicine 2011 6 1415 1427 21796244 10.2147/IJN.S19491 PMC3141869

[B10] Xiao RZ Zeng ZW Zhou GL Wang JJ Li FZ Wang AM Recent advances in PEG-PLA block copolymer nanoparticles Int J Nanomedicine 2010 5 1057 1065 21170353 10.2147/IJN.S14912 PMC3000205

[B11] Leroux JC Allemann E Jaeghere FD Doelker E Gurny R Biodegradable nanoparticles – from sustained release formulations to improved site specific drug delivery J Control Release 1996 39 339 350 10.1016/0168-3659(95)00164-6

[B12] Egusquiaguirre SP Igartua M Hernández RM Pedraz JL Nanoparticle delivery systems for cancer therapy: advances in clinical and preclinical research Clin Transl Oncol 2012 14 83 93 10.1007/s12094-012-0766-6 22301396

[B13] Basile L Pignatello R Passirani C Active targeting strategies for anticancer drug nanocarriers Curr Drug Deliv 2012 9 255 268 10.2174/156720112800389089 22452402

[B14] Ambasta RK Sharma A Kumar P Nanoparticle mediated targeting of VEGFR and cancer stem cells for cancer therapy Vasc Cell 2011 3 26 10.1186/2045-824X-3-26 22082307 PMC3226586

[B15] Choi KY Saravanakumar G Park JH Park K Hyaluronic acid-based nanocarriers for intracellular targeting: interfacial interactions with proteins in cancer Colloids Surf B Biointerfaces 2012 99 82 94 22079699 10.1016/j.colsurfb.2011.10.029 PMC3306773

[B16] Bae YH Interview with Dr You Han Bae: ligand-mediated versus ‘passive’ targeting approaches in nanoparticle oncology research Ther Deliv 2012 3 933 936 10.4155/tde.12.62 22946427

[B17] Danquah MK Zhang XA Mahato RI Extravasation of polymeric nanomedicines across tumor vasculature Adv Drug Deliv Rev 2011 63 623 639 10.1016/j.addr.2010.11.005 21144874

[B18] Kwon G Suwa S Yokoyama M Okano T Sakurai Y Kataoka K Enhanced tumor accumulation and prolonged circulation times of micelle-forming poly (ethylene oxide-aspartate) block copolymer-adriamycin conjugates J Control Release 1994 29 17 23 10.1016/0168-3659(94)90118-X

[B19] Marois Y Zhang Z Vert M Beaulieu L Lenz RW Guidoin R In vivo biocompatibility and degradation studies of polyhydroxyoctanoate in the rat: a new sealant for the polyester arterial prosthesis Tissue Eng 1999 5 369 386 10.1089/ten.1999.5.369 10477858

[B20] Babinot J Guigner JM Renard E Langlois V A micellization study of medium chain length poly(3-hydroxyalkanoate)-based amphiphilic diblock copolymers J Colloid Interface Sci 2012 375 88 93 10.1016/j.jcis.2012.02.042 22424814

[B21] Zhang C Zhao L Dong Y Zhang X Lin J Chen Z Folate-mediated poly(3-hydroxybutyrate-co-3-hydroxyoctanoate) nanoparticles for targeting drug delivery Eur J Pharm Biopharm 2010 76 10 16 10.1016/j.ejpb.2010.05.005 20472060

[B22] Otagiri M Study on binding of drug to serum protein Yakugaku Zasshi 2009 129 413 425 10.1248/yakushi.129.413 19336995

[B23] Singla AK Garg A Aggarwal D Paclitaxel and its formulations Int J Pharm 2002 235 179 192 10.1016/S0378-5173(01)00986-3 11879753

